# Positive parenting program for attention deficit hyperactivity disorder: maternal perspective shifts and child behavior problems reduction in a clinical trial

**DOI:** 10.1186/s13034-025-00960-y

**Published:** 2025-11-19

**Authors:** Nadia Amro, Latefa Ali Dardas

**Affiliations:** 1 Palestine Polytechnique University, Hebron, Palestine; 2 TheUniversity of Jordan, School of Nursing, Amman, 11942 Jordan

**Keywords:** ADHD, Triple p, Parenting intervention, Randomized controlled trial, Palestine, Parenting self-efficacy, Child behavior, Feasibility

## Abstract

**Purpose:**

To evaluate the feasibility and effectiveness of the culturally adapted Triple P intervention in improving parenting competence and reducing behavioral symptoms among children with ADHD in Palestine.

**Methods:**

A randomized controlled trial was conducted with 64 Palestinian mothers of children aged 5–13 diagnosed with ADHD. Participants were randomly assigned to either the intervention group (Triple P) or a control group receiving standard care. Pre- and post-intervention assessments were conducted using the Parenting Sense of Competence (PSOC) scale, the Parenting Scale (PS), and the Strengths and Difficulties Questionnaire (SDQ). Feasibility was assessed through retention rates, session adherence, cultural congruence, and participant satisfaction.

**Results:**

Feasibility findings indicated high session attendance, strong participant engagement, and positive reception of the program, including its online adaptation. Post-intervention, the intervention group reported significantly higher parenting competence (PSOC) compared to the control group. There was also a significant reduction in lax parenting practices and an increase in children’s prosocial behavior. Hyperactivity symptoms showed marginal improvement.

**Conclusion:**

The culturally adapted Triple P intervention was both feasible and effective in enhancing parenting skills and improving child behavior among Palestinian families affected by ADHD. Findings support the integration of Triple P into national mental health services and highlight the importance of culturally responsive, evidence-based interventions in low-resource and conflict-affected settings.

*Trial Registration* Submitted to (ClinicalTrials.gov) on 06/21/2025. Registration number NCT07069621.

## Key Practitioner Message

What is known?

ADHD Children experience significant behavioral difficulties, international Parenting interventions, such as the Triple P (Positive Parenting Program), are effective in enhancing parenting self-agency and reducing child behavioral problems. However, its implementation, feasibility and effectiveness are undocumented in low-resource, conflict contexts like Palestine.

What is new?

The randomized controlled trial demonstrated the feasibility of the culturally adapted Triple P intervention for Palestinian mothers of ADHD children with a significant improvement in mothers’ sense of competence and attitude, with a reduction in child problematic behaviors, and enhanced children’s prosocial behavior.

What is significant for clinical practice?

Health care Practitioners working with ADHD children in low-resource and conflict regions should consider integrating culturally adapted Triple P for parents as part of the management plan to enhance parenting skills and improve child outcomes and family well-being.

## Background

Attention Deficit Hyperactivity Disorder (ADHD) is a prevalent neurodevelopmental disorder that affects approximately 5% of children globally [1], with symptoms often persisting into adolescence and adulthood, impairing academic, social, and occupational functioning [2]. In the Middle East and North Africa (MENA) region, the prevalence is notably higher, reaching 10.3% according to a recent meta-analysis [3]. ADHD diagnosis typically involves clinician assessments supported by parent and teacher reports, alongside standardized rating scales [4]. According to a systematic review and meta-analysis study in Saudi Arabia, the frequency of ADHD ranged from 3.3 to 16.1%. Based on the meta-analysis, the estimated pooled prevalence was 9.95% [5].

Effective management of ADHD increasingly emphasizes behavioral interventions. The American Academy of Pediatrics (AAP) recommends parent training in behavior management (PTBM) as a first-line treatment [6]. Behavioral Parent Training (BPT) equips parents with strategies such as positive reinforcement, structured discipline, and problem-solving, promoting improved child behavior and parental competence. These approaches draw on social learning theory, self-regulation theory, and applied behavior analysis [7].

Among BPT programs, the Triple P – Positive Parenting Program is one of the most widely implemented, offering multi-level interventions tailored to community and individual needs [8]. Developed at the University of Queensland, Triple P aims to enhance parenting knowledge, confidence, and self-regulation while fostering child well-being through supportive environments, assertive discipline, and realistic expectations [9, 10]. Evidence from diverse contexts—including Singapore, Sweden, and Pakistan—demonstrates its effectiveness in reducing child behavior problems, parenting stress, and parental mental health symptoms while strengthening parent-child relationships and promoting long-term positive outcomes [11, 12, 13, 14, 15, 16, 17, 18].

Despite this global evidence, little is known about the applicability and impact of Triple P in Palestine, a region marked by high ADHD prevalence and unique sociocultural challenges. In a recent study, it was found that the frequency of ADHD signs among Palestinian school-age children was (8.7%) [19]. There were no significant differences in prevalence among Arabs and Jews of 18.7%and 17.8% respectively [20].ADHD can profoundly impact family dynamics, with research indicating that mothers of children with ADHD often experience heightened feelings of anger, anxiety, and depression, feeling less effective and successful compared to mothers of children without ADHD [21]. Moreover Mothers of children with ADHD are facing high burden of care and specifically in the region of Hebron [22, 23].Adding to this burden the complex regional political and socioeconomical difficulties facing these families.

Parenting programs that are effective in Western or high-resource contexts may not directly translate to Palestinian families facing chronic stress, limited mental health resources, and cultural barriers to help-seeking. Moreover, few studies have explored the maternal perspective specifically, despite mothers often being primary caregivers for children with ADHD.

This study addresses this critical gap by evaluating the impact of the Triple P program among Palestinian mothers of children with ADHD. Specifically, it examines the program’s feasibility and cultural acceptability, its effects on maternal attitudes and parenting competence, and its efficacy in reducing ADHD-related behavioral symptoms in children. Ultimately, the study aims to inform the development of culturally sensitive, evidence-based interventions for ADHD in the Palestinian context.

## Methods

### Study design and setting

This study employed a randomized controlled trial (RCT) design with pre-test and post-test assessments to evaluate the effectiveness of the Triple P – Positive Parenting Program among Palestinian mothers of children diagnosed with ADHD. The trial was conducted at Hebron Mental Health Center for Children, a governmental facility located north of Hebron. The center offers comprehensive services, including psychological support, speech therapy, behavioral interventions, and pharmacological treatment for children with mental and behavioral disorders.

### Participants and recruitment

Potential participants were identified from ADHD care centers across Palestine. Eligibility criteria included: [1] being the biological mother of a child aged 6–13 years formally diagnosed with ADHD and currently attending a child mental health clinic; and [2] not receiving parental support services from other professional agencies. Exclusion criteria encompassed children with neurological or medical disorders, intellectual disabilities, psychosis, bipolar disorder, or severe aggressive behaviors.

Following ethical approval from the (Arab American University of Palestine) Institutional Review Board and authorization from the Palestinian Ministry of Health, eligible participants were contacted and invited to participate. Informed consent was obtained, and eligibility was confirmed through structured interviews and review of clinical records. Participants were assured of anonymity, voluntary participation, and the right to withdraw at any stage. All data were stored securely in password-protected digital folders and locked physical cabinets accessible only to the principal investigator.

### Randomization and sample size

After confirming eligibility, participants were randomly assigned to the intervention (Triple P) or control group using a computer-generated randomization procedure in Microsoft Excel—RANDBETWEEN () function generated random numbers, between specified values. Participants were assigned to either the control or intervention group based on the rising order of these random numbers.

**Fig. 1:**
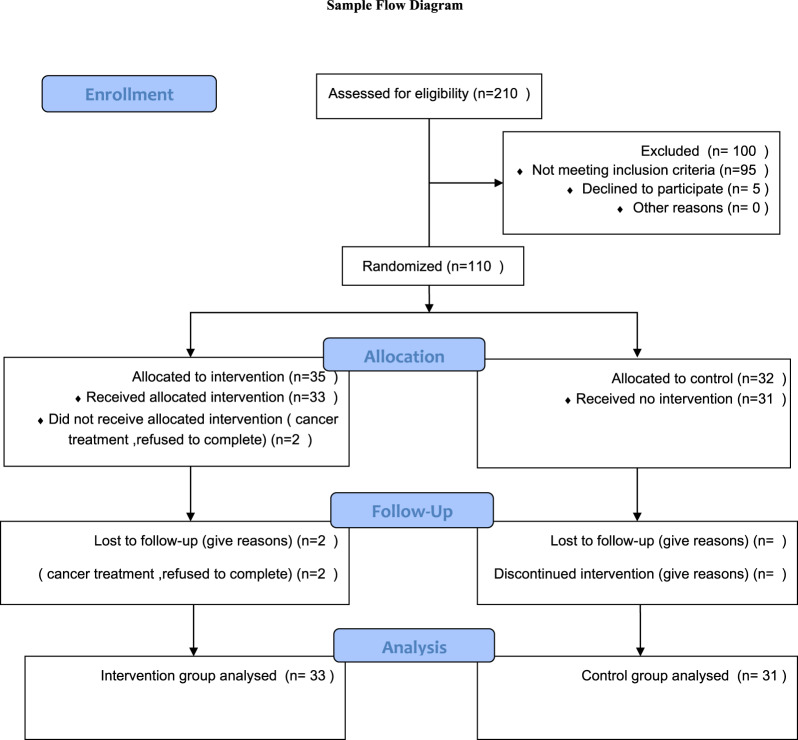
Consort flow diagram

Participants were given sealed envelopes for allocation concealment; the envelopes were opened after the baseline assessment was completed. Participants were recruited between June 2023 and August 2024, sorted in ascending order to achieve a 1:1 allocation. Thirty-three participants were assigned to the intervention group and 31 to the control group (see Fig. 1).

Sample size estimation was based on prior Triple P studies demonstrating moderate to large effect sizes [14, 16]. Using G*Power with an alpha level of 0.05, power of 0.80, and an anticipated effect size of 0.40, the required sample size was 66 participants (33 per group).

### Intervention: triple P – positive parenting program

The Group Triple P Level 4 intervention was culturally adapted for the Palestinian context. The lead researcher completed a certified online training course through the Triple P Association (UK) and received accreditation to deliver the Level 4 program tailored for children with special needs. Core content was sourced from the official Triple P provider materials and Every *Parent* manual, which was translated into Arabic. Supplementary Arabic-language videos from the Triple P platform were used to enhance participant comprehension and engagement. The intervention was administered with fidelity, and according to the original manual checklist, began with an introductory session that introduced the program, obtained written informed consent, built rapport, and collected baseline demographic data and pre-intervention measures. The program was delivered across multiple group sessions (each lasting approximately two hours), incorporating instructional content, video modeling, group discussions, and structured activities (see Table 1). The adherence record indicated 85% of mothers attended at least 6 sessions out of 8. Following the group sessions, each mother received two to three individualized follow-up phone calls (30–50 min each) to support practical application, troubleshoot challenges, and reinforce learned strategies.

**Table 1:**
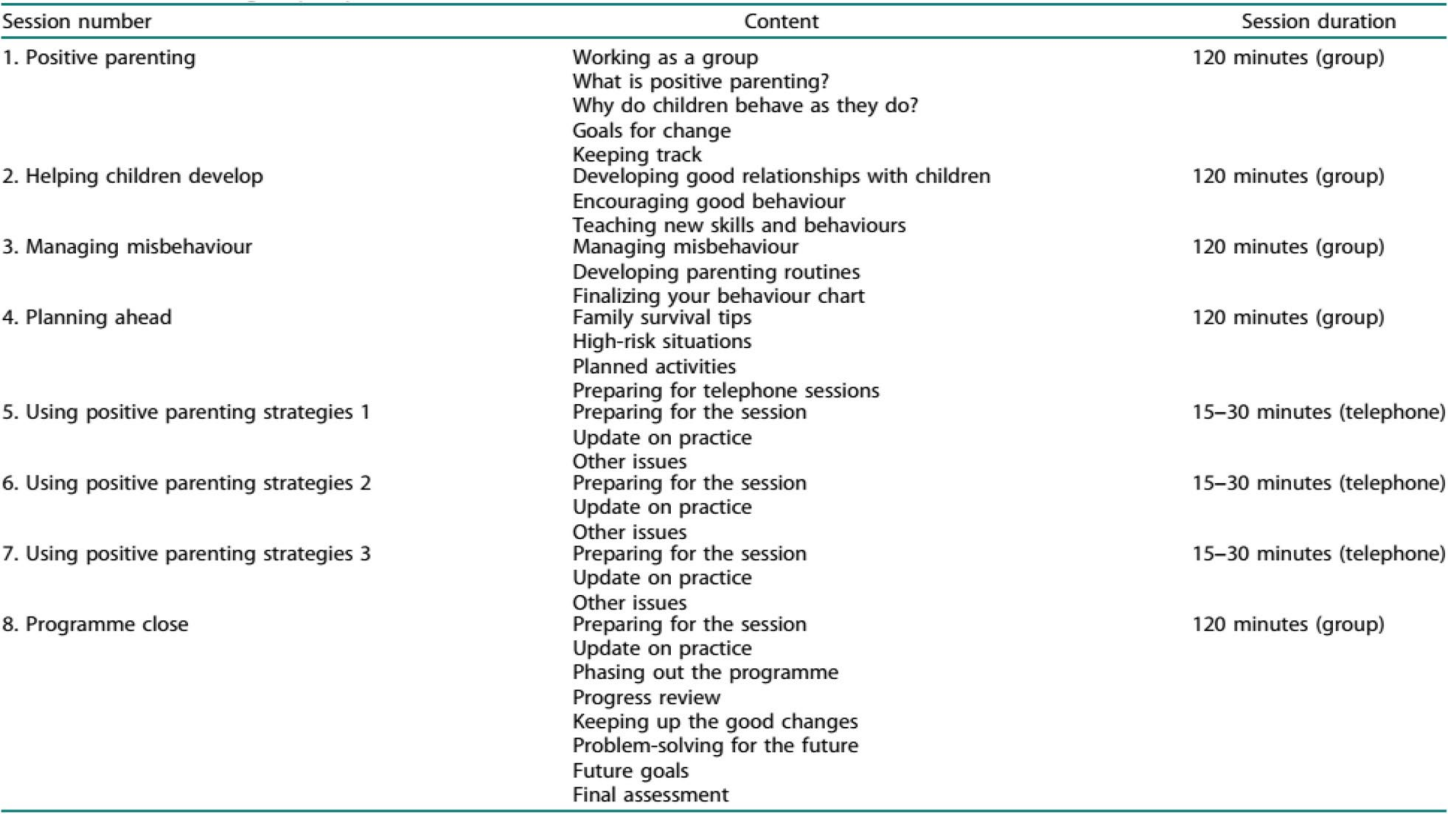
Contents of group triple p sessions adapted from [17]

Ongoing support was provided via a dedicated WhatsApp group, where participants received daily reinforcement through brief messages and multimedia content. These prompts encouraged reflection, reinforced parenting strategies, and supported the development of parental self-efficacy in managing disruptive behavior. Participants were instructed to unenroll in other parenting interventions until completion of the follow-up assessments.

### Control condition

The control group received standard care, which typically included routine clinical follow-ups and general psychosocial support but no structured parenting intervention. Control group participants completed the same pre- and post-assessments as the intervention group, with a two-month interval between measurements. Participants in both groups continued to receive Concomitant Care.

### Outcome assessment

The effectiveness of the Triple P – Positive Parenting Program was evaluated using three validated instruments: two assessing maternal outcomes and one measuring child behavioral outcomes, all based on maternal report.

1. Strengths and difficulties questionnaire (SDQ)

The Arabic version of the Strengths and Difficulties Questionnaire (SDQ) was used to assess children’s emotional and behavioral functioning, as reported by their mothers. Originally developed by [24], the SDQ comprises 25 items spanning five subscales: hyperactivity/inattention, emotional symptoms, peer problems, conduct problems, and prosocial behavior, scores for Prosocial behavior less than 4 is considered abnormal, scores of 7–10 of Hyperactivity considered abnormal; while for Emotional subscale 5–10 is abnormal; as well as 4–10 score is abnormal for Conduct Problems and Peer Problem; the Total difficulties of 17–40 considered abnormal; Total Impact Score exceeding 10 would also be abnormal. It is validated for children aged 3 to 16 years. Previous studies have demonstrated adequate psychometric properties, with a Cronbach’s alpha of 0.73 for the subscales and 0.78 for the total difficulties score [25, 26]. In the current study, internal consistency for the SDQ total score was excellent (Cronbach’s α = 0.88). Test-retest reliability, measured using the intraclass correlation coefficient (ICC) via a two-way mixed-effects model, yielded an ICC of 0.88 (95% CI [0.83, 0.91]).

2. Parenting scale (PS)

The abbreviated 7-item Parenting Scale (PS) was employed to assess dysfunctional parenting practices, particularly discipline styles. It includes two subscales: laxness (three items) and over-reactivity (four items), the cut-offs of clinical stage are ≥ 4.0 for Over-reactivity, and ≥ 3.6 for Laxness, responses rated on a seven-point Likert scale [27, 28]. This tool has demonstrated good psychometric properties, with prior research reporting Cronbach’s alpha values ranging from 0.60 to 0.79 [29]. In this study, internal consistency was moderate (Cronbach’s α = 0.62). The ICC was 0.619 (95% CI [0.431, 0.766]), indicating moderate test-retest reliability across pre- and post-intervention measurements.

3. Parenting sense of competence scale (PSOC)

The Parenting Sense of Competence Scale (PSOC) was used to evaluate parental self-esteem, comprising 17 items that measure two domains: satisfaction and efficacy, a higher score indicates a higher sense of competency. Originally developed by [30] and revised by [31], the scale has shown robust internal reliability, with previous alpha values of 0.75 for Satisfaction, 0.76 for Efficacy, and 0.79 for the total score. The Arabic version was developed using forward and backward translation by bilingual experts. In this study, the PSOC demonstrated good reliability, with an ICC of 0.735 (95% CI [0.630, 0.821]) using a two-way mixed-effects model.

### Data analysis

All statistical analyses were conducted using IBM SPSS Statistics version 27. Data were first screened for completeness and accuracy. Missing data were assumed to be random of less than 3%, and were excluded using listwise deletion. Descriptive statistics (frequencies, percentages, means, and standard deviations) were used to characterize demographic variables and baseline measures. Reliability for each outcome instrument was assessed using Cronbach’s alpha coefficients and intraclass correlation coefficients (ICC). Normality of continuous variables was evaluated using the Kolmogorov-Smirnov test. To assess baseline equivalence between the intervention and control groups, independent samples *t*-tests were performed on all pre-intervention variables. The effectiveness of the Triple P intervention was analyzed by comparing pre- and post-intervention scores within and between groups using independent *t*-tests. Statistical significance was determined at an alpha level of 0.05 (two-tailed). The primary analyses focused on evaluating changes in parenting practices, parental competence, and child behavior. Differences in mean change scores between the two groups were used to assess the impact of the intervention relative to standard care. Sensitivity or subgroup analysis was not conducted.

## Results

### Participant characteristics

The final sample included 64 children diagnosed with ADHD and their mothers. The children had a mean age of 8.00 years (SD = 2.13), ranging from 5 to 13 years. The mothers’ mean age was 36.75 years (SD = 7.54), with ages ranging from 23 to 53 years. The majority of children were male (68.75%, *n* = 44). Most participating mothers were married (90.6%, *n* = 58), while a smaller proportion were separated (6.3%, *n* = 4) or divorced (3.1%, *n* = 2). Regarding educational background, 28.1% of mothers (*n* = 18) held a Bachelor’s degree. The majority were unemployed (70.3%, *n* = 45). In terms of healthcare access, 34.4% of children (*n* = 22) received ADHD treatment through governmental clinics. Most children (75.0%, *n* = 48) were enrolled in mainstream schools. (Table 2).

**Table 2:** Participants characteristics (*N* = 64)

Characteristics	Control group (*n* = 31)	Intervention group (*n* = 33)
Age		
Child’s age in years, mean (SD)	8.81 (2.07%)	8.7 (2.19%)
Mother’s Age in years, mean (SD)	39.7 (8.09%)	34.7 (7.16%)
Marital status		
Married	28 (90.3%)	30 (90.9%)
Divorced	0 (0.0%)	2 (6.1%)
Separated	3 (9.7%)	1 (3.0%)
Mother’s Education Level		
Uneducated	2 (6.5%)	0 (0.0%)
Primary	2 (6.5%)	2 (6.1%)
Secondary	8 (25.8%)	7 (21.2%)
Tawjihi	3 (9.7%)	7 (21.2%)
Bachelor	10 (32.3%)	8 (24.2%)
Diploma (2 years)	5 (16.1%)	6 (18.2%)
Higher studies	1 (3.2%)	3 (9.1%)
Mother employment status		
Yes	11 (35.5%)	8 (24.2%)
No	20 (64.5%)	25 (75.8%)
Child’s gender		
Male	21 (67.7%)	20 (60.6%)
Female	10 (32.3%)	13 (39.4%)
Other diagnosed children		
Yes	2 (6.5%)	11 (33.3%)
No	29 (93.5%)	22 (66.7%)
Type of ADHD treatment center		
Governmental clinic	13 (41.9%)	9 (27.3%)
Specialized center	3 (9.7%)	15 (45.5%)
Private clinic	10 (32.3%)	4 (12.1%)
Not affiliated	5 (16.1%)	5 (15.2%)
School mainstreaming		
Yes	22 (71.0%)	26 (78.8%)
No	9 (29.0%)	7 (21.2%)

### Feasibility results

Feasibility was assessed through indicators including participant satisfaction, data completeness, cultural congruence, retention rates, and protocol adherence. The results demonstrated high feasibility, with strong maternal acceptance of the Triple P intervention. Most participants attended all sessions, despite logistical challenges such as transportation difficulties, financial constraints, and regional instability. Retention was high, with minimal dropout throughout the program. The intervention was culturally well-received, tolerated with no harm, with successful implementation among mothers who shared a common linguistic, religious, and sociocultural background. Additionally, the online delivery of some sessions proved feasible and acceptable for many participants, offering an alternative format when in-person meetings were not possible due to safety concerns or connectivity issues.

### Primary outcome measures

Pre- and post-intervention assessments were conducted for both the intervention and control groups on all study measures. Independent samples t-tests were used to examine group differences at baseline and post-intervention (Table 3). All assumptions for parametric testing—including normality and homogeneity of variance—were confirmed before analysis. The independent variable was group assignment (intervention vs. control), while the dependent variables were the continuous outcome scores from the PS, PSOC, and SDQ.

**Table 3:** Pre and post differences in PSOC, PS, SDQ scales among intervention and control groups

Measure	Intervention (M ± SD)	Control (M ± SD)	Total (M ± SD)	t-value	*p*-value
SDQ-Emotional Problems (Pre)	4.56 ± 2.45	5.16 ± 2.38	4.88 ± 2.42	− 0.983	0.32
SDQ-Emotional Problems (Post)	4.94 ± 2.60	5.16 ± 2.24	5.06 ± 2.41	-0.36	0.716
SDQ-Conduct Problems (Pre)	3.91 ± 1.78	4.39 ± 2.58	4.16 ± 2.22	− 0.863	0.39
SDQ-Conduct Problems (Post)	3.59 ± 1.86	4.48 ± 2.42	4.04 ± 2.18	-1.639	0.10
SDQ-Hyperactivity (Pre)	8.22 ± 1.98	7.71 ± 2.04	7.96 ± 2.01	-1.849	0.06
SDQ-Hyperactivity (Post)	6.78 ± 2.70	7.97 ± 2.01	7.38 ± 2.42	-1.976	0.05*
SDQ-Peer Problems (Pre)	4.38 ± 1.88	3.84 ± 1.51	4.10 ± 1.72	1.24	0.21
SDQ-Peer Problems (Post)	3.97 ± 1.71	3.90 ± 1.76	3.94 ± 1.73	0.150	0.881
SDQ-Prosocial (Pre)	7.47 ± 2.29	6.42 ± 2.81	6.92 ± 2.57	1.627	0.109
SDQ-Prosocial (Post)	7.72 ± 2.20	6.19 ± 2.57	6.98 ± 2.44	2.529	0.01*
SDQ-Impact Score (Pre)	6.77 ± 4.43	5.19 ± 3.27	6.00 ± 3.90	1.598	0.115
SDQ-Impact Score (Post)	6.22 ± 4.24	5.06 ± 3.32	5.67 ± 3.83	1.201	0.234
SDQ-Total Score (Pre)	19.78 ± 5.56	21.32 ± 6.09	20.61 ± 5.82	-1.049	0.298
SDQ-Total Score (Post)	19.28 ± 6.71	21.52 ± 5.54	20.42 ± 6.13	-1.439	0.155
Parenting Scale - Laxness (Pre)	3.0 ± 1.2	3.8 ± 1.0	6.85 ± 2.35	-2.510	0.01*
Parenting Scale - Laxness (Post)	3.3 ± 1.4	3.9 ± 0.9	15.94 ± 4.80	-2.27	0.04*
Parenting Scale - Over reactivity (Pre)	4.0 ± 1.3	3.9 ± 1.0	15.19 ± 4.12	0.451	0.65
Parenting Scale - Over reactivity (Post)	3.6 ± 1.3	3.7 ± 0.9	14.94 ± 4.60	-0.324	0.74
Parenting Scale - Total (Pre)	3.77 ± 0.87	3.96 ± 0.76	3.86 ± 0.81	-0.91	0.36
Parenting Scale - Total (Post)	3.22 ± 0.86	3.53 ± 0.63	3.38 ± 0.76	-1.6	0.11
PSOC - Total (Pre)	63.44 ± 6.97	65.03 ± 8.38	64.22 ± 7.63	− 0.822	0.4
PSOC - Total (Post)	68.28 ± 9.67	62.80 ± 8.81	65.67 ± 9.44	2.3	0.02*

*M* mean, *SD* = standard deviation, *t* = t-value, *p* p-value

### Child behavioral outcomes: SDQ

At baseline, there were no statistically significant differences between the two groups in total SDQ scores, with the intervention group scoring M = 19.78 (SD = 5.56) and the control group M = 21.32 (SD = 6.09), *t* (61) = -1.05, *p* > .05. Similarly, post-test scores remained nonsignificant (intervention: M = 19.28, SD = 6.71; control: M = 21.52, SD = 5.54), *t* (61) = -1.44, *p* > .05. Analysis of the SDQ subscales revealed several notable patterns. For emotional symptoms, no statistically significant differences were observed between the intervention and control groups at either pre-test or post-test (*p* > .05). Similarly, conduct problems showed no significant group differences at both time points (*p* > .05). Peer problems also remained statistically non-significant across the two assessments (*p* > .05), indicating no measurable change attributable to the intervention in this domain. However, for hyperactivity-inattention, a marginally significant difference was detected at post-test, with the intervention group reporting fewer symptoms (M = 6.78, SD = 2.70) compared to the control group (M = 7.97, SD = 2.00), *t*(61) = -1.97, *p* = .053. The associated effect size (Cohen’s *d* = 0.48) suggests a moderate positive impact of the intervention on hyperactivity symptoms. In contrast, prosocial behavior showed a statistically significant improvement post-intervention. The intervention group had higher prosocial scores (M = 7.72, SD = 2.20) than the control group (M = 6.19, SD = 2.57), *t*(61) = 2.53, *p* = .014. This difference corresponded to a medium to large effect size (*d* = 0.64), indicating that the intervention enhanced children’s social functioning as perceived by their mothers.

### Parenting practices: parenting scale (PS)

Total PS scores did not significantly differ between groups at baseline (*t* (60) = -0.911, *p* > .05) or post-intervention (*t* (60) = -1.62, *p* > .05). Regarding the laxness Subscale: At pre-test, the intervention group scored significantly higher (M = 3.08, SD = 1.23) than the control group (M = 3.80, SD = 1.01), *t* (60) = -2.51, *p* = .015, with a medium to large effect size (*d* = − 0.73), indicating greater initial permissiveness. Post-test scores revealed a significant reduction in laxness in the intervention group (M = 3.31, SD = 1.45) compared to the control group (M = 3.95, SD = 0.93), *t* (60) = -2.07, *p* = .04, with a moderate effect size (*d* = − 0.51). regarding the over-reactivity Subscale: No significant differences were found between groups at either time point (pre-test: *t* (60) = 0.45, *p* > .05; post-test: *t* (60) = -0.32, *p* > .05).

### Parental competence: parenting sense of competence scale (PSOC)

Baseline total PSOC scores did not differ significantly between groups (intervention: M = 63.44, SD = 6.97; control: M = 65.03, SD = 8.38), *t* (60) = -0.82, *p* > .05. However, post-intervention analysis showed a statistically significant improvement in the intervention group (M = 68.28, SD = 9.67) compared to the control group (M = 62.80, SD = 8.81), *t* (60) = 2.30, *p* = .023. The effect size was moderate (*d* = 0.59), indicating enhanced parental efficacy and satisfaction.

## Discussion

Over the past two decades, behavioral parent training programs have demonstrated significant clinical benefits in enhancing parenting efficacy, reducing stress, and improving child outcomes in ADHD contexts. Despite such evidence, these interventions have rarely been tested in Arab populations, particularly in Palestine. This study marks the first evaluation of Triple P in Palestine and demonstrates the feasibility of culturally adapting and delivering the intervention in a complex sociopolitical environment. Mothers attended sessions with high adherence despite barriers such as stigma, transportation difficulties, financial constraints, and security challenges. These findings suggest that, when culturally tailored, Triple P is acceptable and implementable in low-resource and conflict-affected settings. Feasibility was also enhanced by offering remote options. Some mothers found online delivery more accessible, helping circumvent stigma and logistical constraints—an increasingly relevant format during periods of political instability. These results support previous findings indicating that digital adaptations of Triple P can sustain engagement in socially disadvantaged communities [32, 33].

Post-intervention results showed significant improvements in maternal sense of competence in the intervention group compared to controls. These findings align with previous studies confirming Triple P’s effectiveness in enhancing parenting self-efficacy, especially in populations managing childhood behavioral disorders [13, 18, 34]. Increased parenting confidence is critical, as it is inversely associated with parenting stress and directly correlated with improved child outcomes and healthier parent-child interactions [11, 17, 35, 36]. The intervention also produced improvements in parenting practices. The results showed a significant reduction in permissive discipline (laxness) in the intervention group, while over-reactivity scores showed downward trends. This shift toward more consistent and constructive parenting aligns with literature emphasizing the role of positive discipline in mitigating disruptive child behavior and reducing parental distress [37, 38]. Further, significant improvements were observed in children’s prosocial behaviors post-intervention, as well as marginal reductions in hyperactivity symptoms. These outcomes underscore the broader efficacy of Triple P in promoting adaptive child behaviors, which not only enhance developmental trajectories but also reduce family stress [14, 39, 40]. There were no significant differences in the emotional or conduct subscales; this may be related to the short period of intervention, with more parenting interventions influencing on externalizing behavior than internalizing, as evidenced by previous studies [41]. These results highlight the need to move beyond diagnosis and medication, advocating instead for holistic interventions that empower caregivers with evidence-based tools.

The findings of this study need to be considered in light of their limitations. First, the study used a pre-post design with no long-term follow-up, limiting insight into sustained effects. Second, the sample size was modest, potentially affecting generalizability, although consistent with prior feasibility studies. Third, intervention delivery by a single researcher may have introduced social desirability bias in addition to a lack of blinding and objective observation using the participant-reported questionnaire. Lastly, data collection occurred during the Gaza conflict and lockdowns, which may have influenced participant stress levels and engagement.

lack of blinding, short follow-up period (2 months), and reliance on participant-reported measures without objective observation.

Despite these limitations, the study offers several strengths. It is among the first to evaluate a structured parenting program for ADHD in Palestine. The use of an RCT design, adherence to CONSORT guidelines, and cultural tailoring of session content contribute to its methodological rigor. The intervention’s flexibility in delivery and responsiveness to participants’ needs enhanced feasibility and ecological validity. Finally, following the completion of the study and preliminary findings, control group participants were granted access to the full Triple P intervention materials. These were delivered via a dedicated WhatsApp group with researcher support, ensuring equitable access to the benefits of the program and enabling asynchronous learning and application.

### Implications and recommendations

The results support the integration of Triple P into mental health services targeting ADHD in Palestine. Nurses, educators, and healthcare providers should be trained in delivering culturally adapted parenting programs. Policies should promote parent engagement, normalize behavioral parent training, and establish counseling centers focused on ADHD and caregiver well-being. Future studies should include larger and more diverse samples, incorporate longitudinal follow-up,and examine mediators (e.g., maternal mental health) and moderators (e.g., intervention level, child age) to optimize impact [39, 42]. Inclusion of fathers and exploration of parental ADHD symptoms are also recommended, as these factors influence intervention adherence and outcomes. Embedding Triple P in academic curricula and primary care could ensure long-term sustainability.

## Conclusion

This study provides evidence that Triple P is both feasible and effective in the Palestinian context. The intervention led to meaningful reductions in child hyperactivity, increases in prosocial behavior, and significant improvements in maternal parenting confidence and discipline practices. Integrating Triple P into national mental health strategies could provide critical support for families affected by ADHD and foster resilience among caregivers in conflict-affected settings.

## Data Availability

No datasets were generated or analysed during the current study.

## Abbreviations

ADHD: Attention Deficit Hyperactivity Disorder
MENA: Middle East and North Africa Region
PS: Parenting Scale
PSOC: Parenting Sense of Competency
PTBM: Parent training in behavior management
RCT: Randomized Control Trial
SDQ: Strength and Difficulties Questioner
Triple p: Positive parenting program
